# Applying a complex systems-informed approach to population health interventions: a methodological case-study example of traffic restriction schemes outside schools

**DOI:** 10.1186/s12889-026-27532-9

**Published:** 2026-05-21

**Authors:** Olivia Alliott, David Ogilvie, Lucy Anderson, Richard Patterson, Kate Garrott, Emma Carey, Cornelia Guell, Jenna Panter, the project team

**Affiliations:** 1 University of Cambridge, Institute of Metabolic Science Epidemiology, School of Clinical Medicine, Box 285, Cambridge Biomedical Campus, Cambridge, CB2 0QQ UK; 2 Bristol Medical School, Population Health Sciences, University of Bristol, Bristol, UK; 3 Department of Applied Health Sciences, University of Birmingham, Birmingham, UK; 4 European Centre for Environment and Human Health, University of Exeter Medical School, Penryn, UK

**Keywords:** Complex systems approach, Population health intervention, Traffic restriction schemes, School Streets, Active travel, Causal loop diagrams, Systems archetypes, Qualitative systems methods, Public health evaluation

## Abstract

**Background:**

Population health interventions may change physical, social or fiscal environments to shift health behaviours and often operate within a complex system of interacting factors. Systems methods are frequently suggested as a way to provide an overview of the interactions that determine behaviours or actions and may help to understand how or why interventions might (or might not) change health. However, there are few examples of how these methods have been used to guide empirical evaluations of public health interventions.

**Methods:**

We describe our application of a qualitative complex systems approach within an ongoing evaluation of a population health intervention: traffic restriction schemes, often referred to as School Streets, where motor vehicle access outside schools is restricted at pick-up and drop-off times. This paper presents a methodological case study illustrating how these methods can be applied in practice. We intended to use systems methods across our evaluation; here we describe how we use these methods to build theory. Following the five-step approach outlined by Alvarado et al., (2023), we produced research propositions, systems archetypes and a causal loop diagram to understand the underlying system in which traffic restriction schemes are implemented and their potential impact on school-based active travel.

**Results:**

In bringing together diverse and multi-disciplinary evidence from different stakeholders we identified unanticipated systems interactions such as increased initial tensions and conflicts between users of different active travel modes (e.g. cyclists vs pedestrians). Using causal loop diagrams and systems archetypes, we developed research propositions focused on funding and implementation, safety, habit formation and the potential for unintended consequences.

**Conclusions:**

Taking a complex systems approach has deepened our understanding of how traffic restriction schemes interact with their broader context and helped us develop a guiding theoretical framework for our ongoing evaluation. We intend this work to stimulate discussion, offer insights for future evaluations of population-level health interventions, and encourage public health researchers to adopt systems methods in their own evaluations.

**Supplementary Information:**

The online version contains supplementary material available at 10.1186/s12889-026-27532-9.

## Introduction

### Background to population health interventions

Population health interventions are non-targeted interventions affecting a group of individuals, community, or population irrespective of people’s health status or engagement in health behaviours [1]. They may change the wider environments-- economic, digital, physical, and social, -- that shape behaviour, complementing ‘high-risk’ approaches which target individuals at elevated risk of poor health [2]. Examples include clean air zones, smoking bans and sugar-sweetened beverage taxes [3, 4].

Evaluating these interventions is challenging as their implementation is often not within the researcher’s control, traditional study designs such as randomised controlled trials are often unsuitable or impractical [1], and therefore establishing causality is more problematic [5, 6]. The difficulty in evaluating these interventions has led to an ‘evaluative bias’ where the evidence favours individual behavioural interventions, which can be evaluated using traditional study designs [1, 7]. To combat this, researchers are increasingly using natural experimental methods, which evaluate the effects of an intervention in a real-world setting without randomisation [8]. However, greater awareness and use of diverse methods are needed to estimate intervention effects and to understand how, where, and for whom these effects occur [8]. In their recently published framework, Craig et al. highlight advances in the design and planning of natural experiment evaluations, including the importance of a systems perspective, mixed methods and stakeholder involvement. [8] To generate robust and actionable evidence for public health policy, we need natural experimental approaches that account for the broader systems and contexts in which interventions take place [1, 9].

### Taking a complex system approach to the evaluation of population health interventions

Systems approaches have been advocated to understand the interacting factors influencing the impact of population health interventions [5, 10]. A *complex system *is characterised by interrelated parts whose interactions create emergent properties which cannot be fully understood by examining the components individually [5]. While evaluation research has begun to address this complexity, much of the focus remains on the intervention itself (e.g. its features and targeted behaviours) rather than the broader system in which it operates [11].

A complex systems approach looks at the system as a whole, considering “people, processes, activities, settings, and structures, and the dynamic relationships between them,” [10] and how systems and interventions adapt to each other [6]. Systems methods have traditionally been used to map system-level influences on behaviours, rather than to evaluate intervention effects [12]. When used in evaluation, complex systems approaches can address theory-driven questions, including theories of change, intervention mechanisms and differential impacts across subgroups, moving beyond linear conceptual frameworks [12]. They support the growing shift toward practice-based evidence, evaluating interventions as implemented [6, 12, 13].

However, the use of complex systems perspectives within natural experimental evaluations is still emerging. Uncertainty remains around how to apply these approaches, which methods to use, and what types of evidence they produce [5, 6, 14].

### What would a systems informed approach look like in the evaluation of a population health intervention?

‘Systems methods’ is a broad term used to refer to methodological tools and techniques applied when taking a systems approach [12]. A variety of methods are employed to understand complex systems. These range from quantitative methods using mathematical and simulation tools such as agent-based modelling and network analysis, which examine how systems function dynamically and predict future outcomes [15], to qualitative methods such as concept maps and causal loop diagrams (CLDs) which represent a system’s structure, identify leverage points or explore the potential for unintended consequences [16].

The dominant approach often depends on the research objective, as well as existing evidence and knowledge about the specific intervention being evaluated [17]. Broadly speaking, quantitative methods are often more effective for testing and predicting the impact of interventions on a large scale, where there is substantial data available [18]. Qualitative methods are particularly useful during the exploratory phase of an evaluation, particularly when data are limited. They can be used to engage stakeholders to understand complex system structures, to explore the overall dynamics in a system, and to identify potential outcomes as well as those along the causal pathway, or potentially relevant factors to measure in any quantitative analyses [18].

Practical examples of the application of complex systems approaches to evaluating population health interventions are limited [12, 19, 20]. McGill et al. note that while systems methods have been applied to specific phases of evaluation, such as theorising and impact assessment, few studies have used these approaches throughout an entire evaluation lifecycle [5]. Similarly, Medical Research Council guidance on complex interventions acknowledges systems thinking but lacks detail on practical application [11].

Typically, systems-oriented evaluation frameworks are designed for scenarios where research teams have some influence over the development and implementation of an intervention or in effecting change within a system [21, 22]. For example, the Evaluation of Programmes in Complex Adaptive Systems (ENCOMPASS) framework was created based on the development and evaluation of a new programme, allowing researchers to shape the intervention from a systems perspective [22]. Garcia et al. propose an "Action-oriented framework for systems-based solutions aimed at childhood obesity prevention," which focuses on envisioning and implementing systems-wide change [21], but that study’s research team had some agency in co-envisioning and co-effecting change within the system. In many evaluations of population health interventions, however, researchers have little control over the design or implementation of the intervention. Alvarado et al. provide methodological guidance for system-informed evaluations where the research team has no control over the intervention or the broader system [6]. Drawing on the above frameworks [22–25], they propose a five-step approach to generating systems-informed research propositions for evaluating large-scale policy or infrastructure interventions using CLDs [6]. After this process, they developed a preliminary conceptual framework for each intervention and a set of systems-informed research propositions to guide future empirical evaluation [5].

### Aim of this paper

This paper presents a methodological case study applying Alvarado et al.’s five-step approach within an ongoing empirical evaluation of a population health intervention, integrating insights from their evaluations and adapting the approach as suggested [19]. As part of a broader programme of work on traffic restriction schemes outside schools in Great Britain, we demonstrate how a complex systems approach can be used to refine and guide data collection and analysis, outlining current progress and future directions. We present this as a practical example to stimulate discussion, highlight opportunities and challenges and inform future evaluations of population-level health interventions.

While this work is embedded within a broader empirical evaluation, the focus of this paper is methodological; we do not report evaluation outcomes but instead illustrate how systems methods can be used to guide evaluation design and theory development.

## Process

### Case study: evaluation of traffic restriction schemes outside schools

To provide a case study example, we focus on our application of a complex system approach to evaluate a specific population health intervention: traffic restriction schemes. These schemes place temporary restrictions on motorised traffic outside schools at the beginning and end of the school day. Exemptions vary between local governments, but often include local residents, people with disabilities, deliveries and emergency services. Even if exempt, local residents are encouraged to avoid travel and limit visitors during these times. In the UK, these are also referred to as ‘School Streets’, ‘No Car Zones’ or ‘School Exclusion Zones’, they aim to promote active travel to improve health and environmental outcomes [26].

These schemes have expanded rapidly across the UK in recent years, driven by the Covid-19 pandemic, policy interest and funding availability [27]. Despite growing implementation and support from local authorities, robust evidence on their impacts, underlying mechanisms, and effectiveness across different contexts remains limited [27]. The Children’s Road Safety Outside School (CROSS) study seeks to address this through the following research questions:

- Do traffic reduction schemes outside schools’ impact children’s levels of active commuting and travel, and are there differential impacts according to geographical area or socio-economic context?
- What are the mechanisms and pathways by which these schemes may (or may not) work to change children’s levels of active travel?
- How might the impacts and mechanisms of these vary by context (e.g., social, physical, school and community) and how might impacts be optimised across contexts?

Our evaluation is structured as four interconnected work packages (WPs). WP0 focuses on (i) identifying mechanisms of the schemes, producing a systems map and a theory of change and (ii) scoping methods for integrating qualitative and quantitative data which can be used to evidence mechanisms [28]. This lays the foundation for searching for and appraising suitable national quantitative data (WP1), conducting an effectiveness analysis (WP2), and qualitative data collection and analysis relating to mechanisms and contextual variations (WP3). The research team includes academic expertise in the design and evaluation of public health interventions, active travel and physical activity, children’s geography, and transport planning, alongside practice partner expertise in the design and implementation of active travel schemes and in advising governments and local authorities.

### Our application of a complex systems approach

We take a qualitative complex systems approach [17] due to the paucity of existing evidence for these schemes, making exploratory evaluation more appropriate. To guide this approach, we applied the process outlined by Alvarado et al. [6] as follows:

1. Represent the underlying system
2. Develop an understanding of the intervention
3. Identify links between the intervention (and its influences/impacts) and the underlying system
4. Generate systems-informed research propositions from the CLD, drawing on systems archetypes where relevant
5. Sense-check the systems-informed research propositions with subject area experts and stakeholders.

Through this process we aimed to develop a CLD that captures the complexity of the system within which traffic restriction schemes operate across different contexts. A CLD is a specific type of system map that focuses on depicting feedback loops and causal relationships within a system [29, 30]. CLDs consist of system elements relevant to the research questions and the causal relationships between them [29]. These abstract concepts, systems, elements, and causal relationships vary depending on the context (definitions of these and other terms we use here are provided in Table 1). At a minimum, CLDs include system elements, their causal connections, and an implicit or explicit system boundary [30, 31].

**Table 1:** Glossary of key term used in complex systems approaches [5]

Complex Intervention	An intervention characterised as complex due to: • “Number of interacting components within the experimental and control interventions • Number and difficulty of behaviours required by those delivering or receiving the intervention • Number of groups or organisational levels targeted by the intervention • Number and variability of outcomes • Degree of flexibility or tailoring of the intervention permitted” [32].
Causal Relationships	Links between elements, where one influences another. These can be: Positive – an increase/improvement in one element causes an increase/improvement in another Negative – an increase/improvement in one element causes a decrease/decline in another These connections can operate over short or long time horizons
Connectivity Analyses	Identifies elements that influence the greatest number of others. This may involve counting primary outbound connections (arrows) or including secondary outbound connections. Highly connected elements can have broad and significant impacts across the system
Causal Loop Diagrams	Visual tools that map out cause-and-effect relationships between system variables to show how they influence one another
Elements	Key system components that interact or influence each other. Every element must be linked to at least one other in the system
Feedback Loop	A recurring cycle where a change in one part of the system either reinforces (amplifies) or balances (reduces) further change
Group Model Building	A collaborative process in which stakeholders collectively map and understand a system by sharing perspectives
Research Propositions	Descriptive hypotheses about how loops and loop combinations function within a system, particularly focusing on feedback, to guide empirical evaluation
System	A network of interconnected entities (e.g., people, organisations, resources) whose interactions produce patterns of behaviour over time
System Archetype	A recurring structure or pattern of behaviour commonly found across different systems
Whole-System Intervention	A coordinated approach aimed at creating change across multiple parts of a system—for example, a local obesity strategy that includes school, high street, governmental, and media interventions

See Kumu for an interactive presentation of application of a complex systems approach (https://Ora26.kumu.io/the-childrens-road-safety-outside-school-bbf70c1d-e442-4423-9259-0256ec21d634).

#### Step 1: represent the underlying system

In step one, Alvarado et al. describe identifying and developing an understanding of the underlying system within which the intervention is introduced [6]. They outline various starting points for representing the underlying system, ranging from the use of existing system maps to the creation of new maps developed through detailed participatory system mapping involving broad consultation, engagement, co-creation, systematic reviews, literature searches, or a combination of these approaches. They note the importance of “trade-offs between various approaches, considering the time and resources required at this stage and subsequent ones” [6] p6.

Due to time and resource constraints, we developed a new map based our own knowledge, empirical studies, systematic reviews and reference and citation tracking, through which we identified 12 relevant papers, including one from grey literature. This included evidence on youth active travel more broadly. We used Panter et al.'s conceptual framework for environmental determinants of children's active travel as a foundation for mapping the underlying system, as this was the most relevant framework among the published literature [33]. This helped us create a map of the underlying system influencing children’s active travel to school, focusing on policy, organisational, environmental and individual-level influences (Appendix 1). This provided a foundation for considering possible interconnections between traffic restriction schemes outside schools and the underlying system concerning school-based active travel.

#### Step 2: develop an understanding of the intervention

Alvarado et al. describe developing an understanding of the intervention using a combination of approaches tailored to the nature of evaluation (e.g. single vs. multi-site) and the availability of existing evidence [6].

We began by assessing the evidence to understand how the schemes might influence school-based travel. This included drawing on learnings from our recent study evaluating London’s Ultra Low Emission Zone (ULEZ), where key stakeholders discussed traffic restriction schemes in the context of London’s ULEZ [34], as well as a grey literature review and qualitative study with local authority officers [26]. We theorised about the proximal and distal impacts of traffic restriction schemes, focusing on direct relationships, potential pathways, and unintended effects (e.g., increased traffic and congestion on nearby streets). We included directional information in the map based on the most plausible relationships and did not distinguish between hypothesised and empirically tested connections, as there was very limited published academic evidence on the schemes supported by data (Appendix 2).

**Fig. 1:**
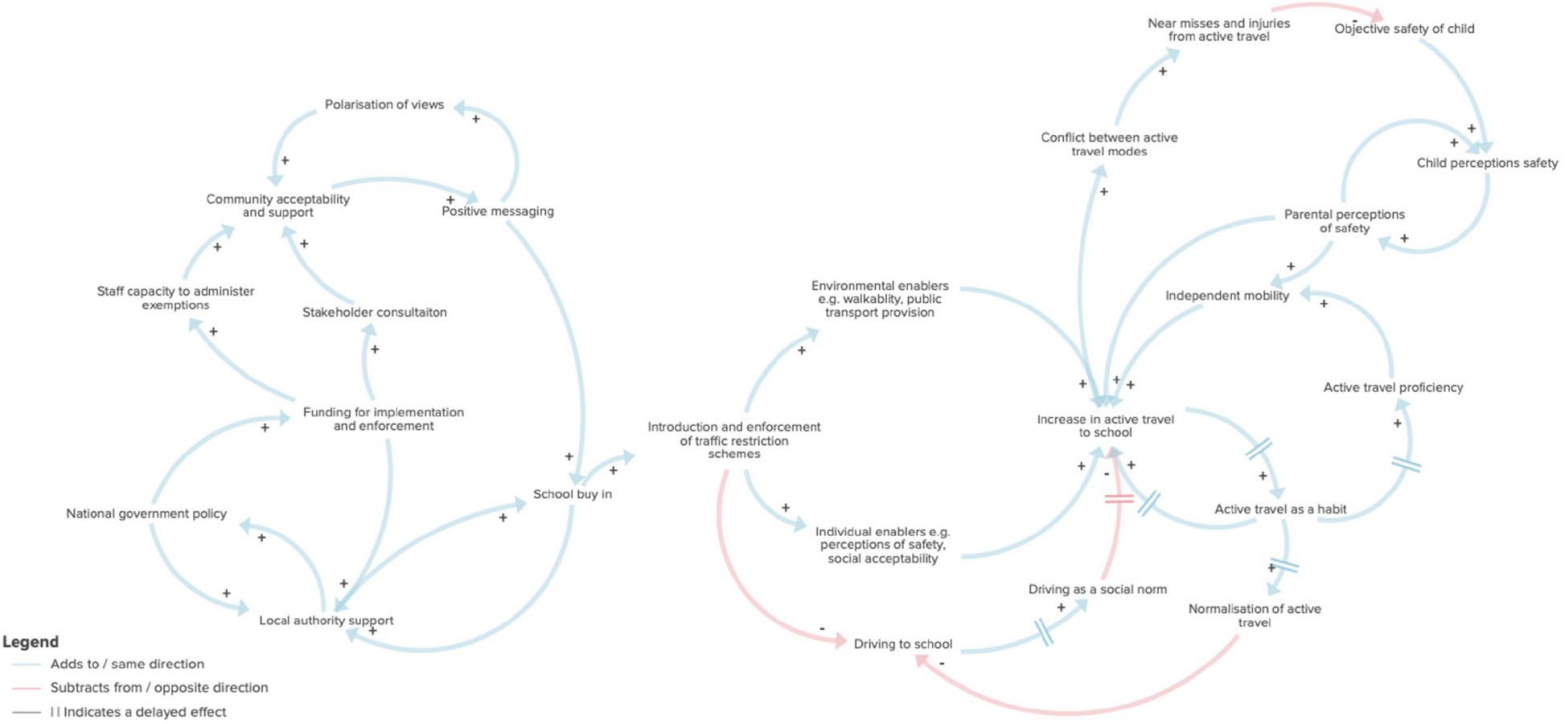
Critical causal pathways by which we hypothesised traffic restriction schemes to work

**Fig. 2:**
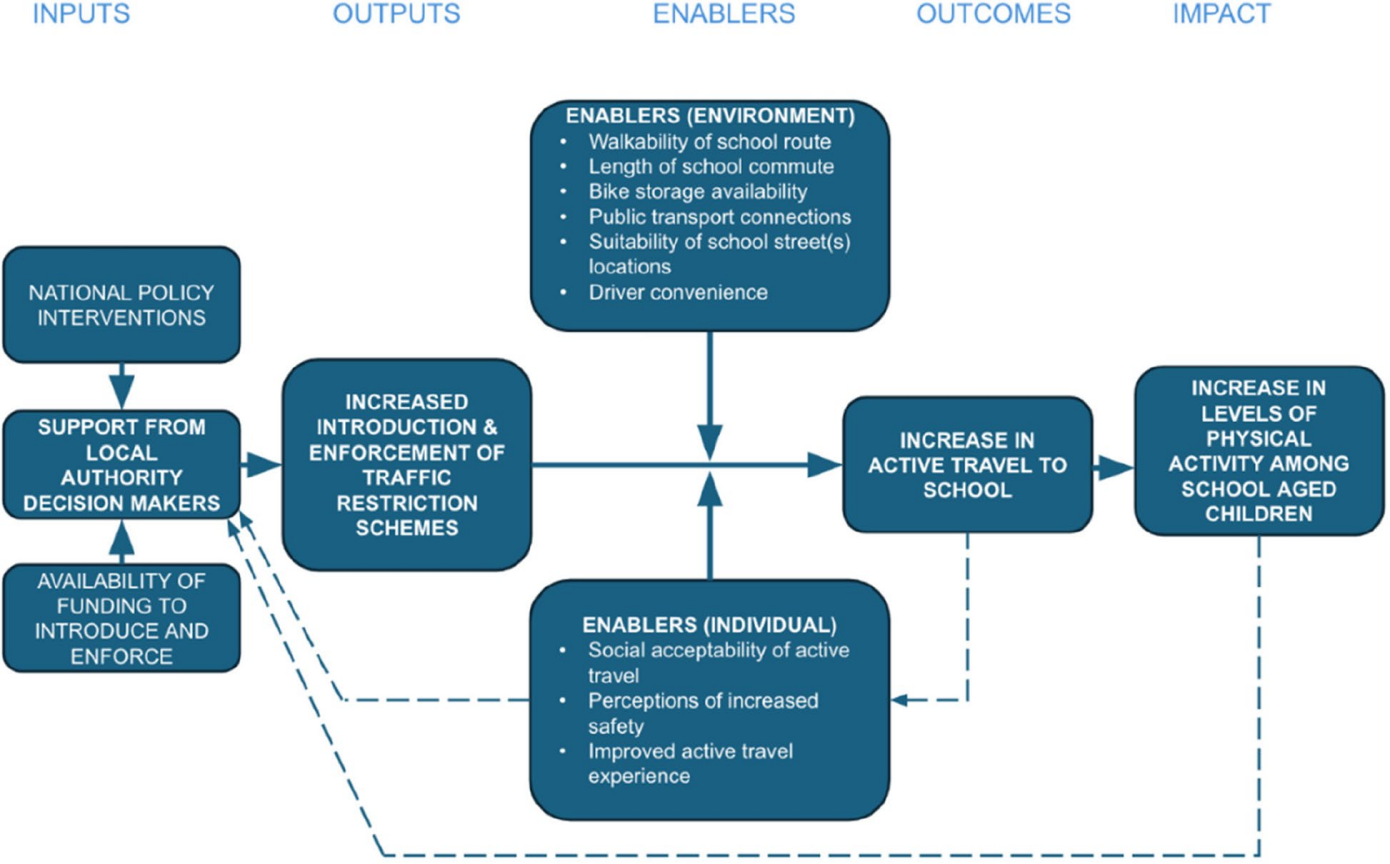
Guiding theory of change model

From this, we developed a simpler, more specific model of the critical causal pathways by which we believe traffic restriction schemes to operate (Fig. 1) and a theory of change model (Fig. 2). This helped us manage the complexity of the broader system, clarify pathways to impact, and situate this approach alongside the more traditional linear models commonly used to guide evaluations. This was particularly helpful in providing a framework for our quantitative analysis (WP2).

#### Step 3: identify links between the intervention (and its influences/impacts) and the underlying system

**Fig. 3:**
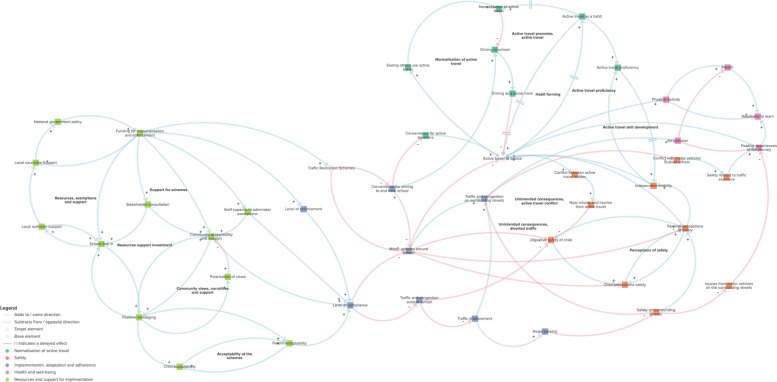
Causal loop diagram

During this stage, Alvarado et al. describe an iterative process connecting the intervention to the underlying system by exploring interconnections and potential feedback loops. [6] Here, we reorganised our elements and added new intermediate steps to make the links explicit (e.g., clearly highlighting the connections between "active travel," "physical activity," and "health"). This stage employed an iterative process to develop a large and detailed system map and to start identifying potential feedback loops (such as habit formation though active travel, shown in the “habit forming” loop of Fig. 3). The process included relabelling and reorganising elements to better connect intervention-related concepts and to consider potential unintended consequences (such as increased illegal parking). At this stage, we developed our conceptual system maps from steps 1 and 2 into a large and detailed CLD (Fig. 3).

We identified 21 causal loops within our diagram with between 2 and 8 connections. We drew on all available evidence (outlined in step 1) using Deegan’s 10-step process for coding causal relationships from text and developing associated causal maps [35].

Aligning with systems methods, included elements had to be amenable to change within the defined system boundary (factors related to the implementation and impacts of traffic restriction schemes), without the capacity to zoom in (e.g., breaking topography down into its physical features) and had to affect or be affected by at least one other element in the system [30]. Elements not directly related to traffic restriction schemes were excluded. Decisions regarding inclusion and exclusion were made iteratively by the research team and informed by stakeholder input during the workshops. During this process, we created a list of “other considerations” highlighted within the research project team, e.g. the number of motor vehicles around the school before implementation. These were factors outside the boundaries of our diagram which we deemed important to our evaluation.

#### Step 4: generate systems-informed research propositions from the CLD

In step 4, Alvarado et al. describe analysing joined-up causal loops to develop refutable systems-informed research propositions. These are narrative descriptions of specific loops or combinations of loops developed through a process akin to reflexive thematic analysis [36], involving iteration between high-level themes and detailed cause-effect relationships. We applied this approach by initially describing smaller combinations of loops, before generating higher-level abstracted CLDs combining initial loops to develop testable, systems-informed research propositions which link the patterns observed in step 3 (Table 2). Alvarado et al. emphasise the benefit of using systems archetypes where relevant. These are recurring patterns of behaviour found in various systems, characterised by common structures and dynamics that lead to predictable outcomes. See Kim & Anderson for a full overview [37].

**Table 2:** Emergent archetypes and corresponding systems-informed research propositions derived from iterative synthesis of joined-up causal loops

Archetype	System-informed research propositions
Archetype 1: Parental perceptions of safety act as a gatekeeper	Parental perceptions of safety play a pivotal role in determining the success of traffic restriction schemes in promoting school-based active travel. Perceived safety acts as a key driver within reinforcing and balancing feedback loops. When traffic restriction schemes lead to initial increases in active travel, children may develop greater proficiency and confidence. This, in turn, can encouraging greater independent mobility, positive perceptions of safety and further reinforce active travel, creating a virtuous cycle Conversely, if parents perceive the environment as unsafe, this can limit children’s independent mobility, dampening active travel uptake and potentially stalling the benefits of the intervention. This dynamic reflects a tension between reinforcing momentum and balancing constraints. Notably, parental and child perceptions are interlinked, each shaping and responding to the other Together, these dynamics highlight the potential importance of fostering positive perceptions of safety among parents to sustain long-term behaviour change and maximise the scheme’s impact This aligns with the “Limits to Growth” archetype, though it exhibits an initial emphasis on reinforcing (positive) feedback processes and shares features with the “Shifting the Burden” archetype [37].
Archetype 2: The normalisation of active travel	Building on archetype 1, increased proficiency in active travel among young people can create a reinforcing loop that supports long-term behaviour change. As young people become more confident and capable, they are more likely to continue travelling actively to school, gradually embedding it as a habitual and socially normalised practice. This shift not only influences individual behaviour but can also shape travel norms, encouraging wider uptake across the school community Over time, this self-reinforcing loop may contribute to a sustained increase in school-based active travel, delivering cumulative benefits for physical activity levels, health and well-being This archetype highlights how normalisation and habit formation can be powerful levers for systemic change, particularly when initial momentum is supported by consistent opportunities and encouragement This aligns with the "Success to the Successful" archetype, with elements of a virtuous cycle reinforced by social normalisation and habit formation [37].
Archetype 3: Ripple effect of traffic restrictions	Architype 3 is an example of a “Fixes that Fail” archetype [37]. It highlights that while traffic restriction schemes may reduce congestion on targeted streets, they could inadvertently increase traffic and congestion on the surrounding streets, as well as illegal parking. This shift could lead to more road traffic accidents in these areas and potentially negate increases in active travel to school achieved through the intervention and impact perceptions of safety. Increased active travel to school might cause a shift in accident patterns as users adapt to new shared spaces, which could also lead to a stabilisation or reduction in active travel rates (linking to the role of perceptions of safety in archetype 1) Together, these feedback loops highlight the importance of anticipating secondary impacts and ensuring that interventions address the wider system, not just the immediate problem This exemplifies the "Fixes that Fail" archetype, highlighting how short-term gains from an intervention may be undermined by unintended side effects elsewhere in the system [37].
Archetype 4: Funding, support, acceptance and polarisation	The implementation of traffic restriction schemes is shaped by a reinforcing cycle between national policy support, funding availability, stakeholder engagement and local uptake. As government investment grows, improved consultation and administrative capacity help align schemes with local needs. This enhances community support, encourages school buy-in and strengthens local authority backing, ultimately reinforcing national policy. This positive feedback reflects a “Success to the Successful” archetype However, a parallel dynamic poses a risk. Efforts to build community support may also trigger opposition if some groups feel excluded or disadvantaged. Rising acceptance could provoke stronger resistance, deepening polarisation and gradually weakening public support. This reflects a “Fixes That Fail” archetype, where short-term gains give way to unintended, long-term setbacks Together, these dynamics highlight the need for inclusive engagement and thoughtful communication to sustain momentum and prevent reinforcing divisions This aligns with a combination of the “Success to the Successful” and “Fixes that Fail” archetypes, showing how reinforcing momentum in policy uptake can be disrupted by unintended social dynamics that generate polarisation [37].

We developed four archetypes based on joined-up causal loops (Table 2). Three of our archetypes did not fit any of the classic templates [37]. We therefore created custom archetypes which borrow elements from several pre-existing archetypes to describe a unique pattern of behaviour. For example, Archetype 1 highlights the latent potential for a reinforcing loop that is constrained by perceptions, drawing on the “Limits to Growth” and “Shifting the Burden” archetypes [37].

#### Step 5: sense-check the systems-informed research propositions with stakeholders

In this final stage, Alvarado et al. describe sense-checking the system-informed research propositions with stakeholders, making changes to reflect their feedback. We took this opportunity to sense-check our CLD, research propositions and resultant archetypes. This process included online and in-person group modelling building (GMB) sessions, as well as one-on-one feedback sessions with our research team, before engaging the broader research community and key stakeholders from policy and practice.

Academic, public and practice stakeholders were invited (*n* = 15) based on their knowledge of the schemes at either policy or implementation level. Participants included academic researchers with expertise in transport, urban planning and public health, representatives from parent and community advocacy groups (e.g., Mums for Lungs), local government officers involved in transport planning, public health and environmental policy, and practitioners working within health services and local authorities who had experience supporting or implementing Traffic Restriction Schemes. These stakeholders were selected to capture perspectives from policy development, implementation and the community.

To support engagement from stakeholders with varying familiarity with systems methods, we developed a short video introducing key system mapping concepts, explaining how to interpret a causal loop diagram, and presenting the draft CLD developed in earlier stages. Stakeholders were invited to review the video before providing feedback. During GMB sessions and follow-up discussions, stakeholders were asked to reflect on relationships in the CLD, identify missing elements, suggest relationships that should be added or removed, highlight potential feedback loops. Stakeholders were also invited to comment on the proposed research propositions and whether these reflected their understanding of how traffic restriction schemes operate in practice.

To accommodate differing availability and preferences, we offered multiple routes for engagement. Some policy stakeholders provided feedback via email due to time constraints. Professional groups, including academics and local practitioners, participated in group model building sessions to discuss and refine the diagram collectively. Community groups were engaged during their existing meeting forums to make participation as convenient and comfortable as possible.

A key theme that emerged from our GMB sessions was the importance of resources and support for implementation. Stakeholders emphasised the role of local capacity, staffing and ongoing support in shaping how schemes were delivered and sustained. This feedback is coded as green nodes on our CLD (Fig. 3). It also led us to update the diagram to include a dedicated section on “resources and support for implementation” (Fig. 3). Additionally, it prompted us to engage a broader range of stakeholders beyond policy and practice, such as parent and community groups, to better understand the acceptability of the schemes and the impact of varying resources and support.

At the end of this process, we had developed a preliminary CLD (Fig. 3) which we will return to throughout the project, alongside systems-informed research propositions, testable archetypes and an intervention framework to guide our future empirical evaluation.

### Current application and future plans

Our CLD, research propositions and systems archetypes will serve as a framework for recognising and analysing patterns of behaviour across various contexts, guiding future systems-informed analyses, and laying the foundation for an evaluation design informed by a systems perspective. These have guided our approach to site scoping and selection, prompting us to consider context-specific factors, e.g. the layout and design of surrounding streets, which might have otherwise been missed. The methods of qualitative system mapping encouraged us to engage with stakeholders, broadening the range of perspectives that informed our approach. This unexpectedly allowed us to leverage their networks and contacts (e.g. regional road safety teams), which facilitated our acquisition of quantitative data and participant recruitment.

Starting with a conceptualisation of the underlying system allowed us to explore potential feedback loops and how these might impact the success of traffic restriction schemes, e.g. the potential for an unintended increase in injuries from active travel negatively impacting future active travel. This differs substantially from common practice in public health, where evaluations are typically based around a linear conceptual framework [6].

In our qualitative work package, our research propositions guided the development of our interview topic guides, including specific lines of questioning around parental perceptions of safety, habit formation and potential unintended consequences. Moving forward, we will look for evidence of these patterns across different contexts using ethnographic observation. In our quantitative work package, which assesses the impact of these schemes on children’s school travel patterns, the systems map helps to remind us about the small part of the system which we are able to test with this data and reinforces the range of complex and interrelated pathways by which these schemes lead to changes in travel patterns. It also highlights where other data might be needed to support evidence of mechanisms which would strengthen the basis for causal claims.

Our research propositions and archetypes will be transformed into site-specific hypotheses which consider different contextual factors, e.g. urban vs rural; and operationalised to reflect varying levels of implementation at different sites, e.g. using enforcement cameras, or co-located park and stride schemes (places to park a car, from which the rest of the journey can be completed using non-motorised modes). For example, an increase in active travel (Archetype 2) in an area without supporting infrastructure, such as segregated cycle lanes and designated crossing points, could lead to increased conflicts between active travellers (Archetype 3). Additionally, areas with lower levels of enforcement may be more affected by unintended consequences, or by drivers ignoring the schemes due to less severe penalties.

A complex systems approach will inform our future qualitative analyses. For example, we will examine if and how these archetypes manifest across different intervention contexts. We also aim to determine whether any of the pathways within the systems map can be substantiated by our data. This can be tested in various ways, integrating both qualitative and quantitative evidence through methods such as process tracing or convergence analysis [28].

## Discussion

We present our initial experiences applying a complex systems approach to an evaluation of traffic restriction schemes implemented outside schools. Following the methodology outlined by Alvarado et al., we have developed a CLD to guide our evaluation, formulated research propositions and resultant archetypes to be tested using both quantitative and qualitative data, and provided a framework for future integration [6, 28]. Our approach was informed by a review of relevant literature, as well as input from key stakeholders outside the research team, including academics with topic expertise and practitioners involved in implementation.

Our systems-informed approach highlighted several key feedback loops and archetypes that deepen our understanding of how traffic restriction schemes may operate in practice. We identified reinforcing loops related to habit formation, whereby improved safety and visibility of active travel modes support sustained behaviour change. In contrast, balancing loops illustrated how limited infrastructure or weak enforcement can undermine intended outcomes, potentially leading to unintended consequences such as conflict between different road users. These dynamics were represented in four archetypes, including latent potential constrained by perceived safety, implementation fragility, and varying impacts across contexts. Collectively, these insights underscore the importance of situating interventions within the broader systems in which they operate. This understanding is shaping our ongoing evaluation by helping to identify context-specific mechanisms and explore how outcomes might be optimised across diverse settings.

Below, we reflect on our process and share key insights.

### Our experience and learning from applying a complex systems approach

There are various starting points for developing a system map, which can be hard to navigate. We took a pragmatic approach, allowing us to closely align the mapping process with our research aims, giving us time to immerse ourselves in the complex systems approach before wider dissemination. Orienting stakeholders to the rules and terminology of this approach during GMB was challenging, but tools such as Scriptapedia proved useful for structuring the sessions [38]. We later developed a short video which helped stakeholders familiarise themselves with a complex systems approach at their own pace. Allocating time and resources to interactive methods such as Miro and Kumu improved engagement and productivity during our sessions. To date, stakeholders have provided positive feedback and expressed interest in staying updated on our findings, underscoring the value of this approach in bridging the well-documented gap between research and practice [39].

We found that taking an iterative process helped us to set the boundaries of our CLD and to represent contrasting views from project stakeholders. In Step 2, shifting our focus from the broader system to the specific intervention was particularly helpful in navigating this challenge. This narrowed our focus and was more aligned with the research team's evaluation background, where it is common to begin with the intervention [23, 40]. Identifying causal loops was challenging, especially when determining the direction of arrows to accurately represent diverse feedback. The iterative process allowed us to account for more distal consequences, potential feedback loops, and unintended outcomes. This process underscores the value of revisiting steps, rather than following a strictly linear approach. Becoming more familiar with the functions of Kumu helped facilitate multiple iterations and in identifying causal loops [30].

Developing systems archetypes enabled us to deepen our understanding of, and critically reflect on, the dynamics depicted in our CLD. It also offered a platform for stakeholders to provide context-specific reflections. This process was valuable in refining our hypotheses for qualitative analysis and in reinforcing the relevance of certain variables already under consideration for selecting control sites in our quantitative analysis. While we encountered difficulty applying common archetypes to the causal structures, our method aligns with Kim’s recommendation to use systems archetypes in a way that offers insight into the specific challenges being addressed [37]. By recognising and reflecting on these archetypes we hope to be better positioned for developing policy recommendations based on recurring patterns in a complex travel system.

This was our first experience applying a complex systems approach to evaluate a population health intervention. We note that other authors have reported variations in the depth and sophistication of system approaches used within public health, often requiring expertise in evaluation, systems thinking, and the specific intervention being evaluated [23]. The structured steps provided by Alvarado et al. guided us through the process, allowing us to build on their insights and those of others [41]. Several resources proved helpful to us, including a complexity-informed seminar series [42], the Systems Evaluation Network (SEN) [43], and the recent NIHR School for Public Health Research Systems Thinking webinar series [44]. We were fortunate to have expertise within our research group, particularly Alvarado and Chavez-Ugalde who established a complex systems working group. These colleagues served as peer mentors and sounding boards, while the broader group provided a platform for presenting and discussing our application of a complex systems approach.

Complex systems approaches may be particularly well suited to the evaluation of active travel interventions, which are typically embedded within multiple interacting systems spanning public health, transport, urban design, education and environmental policy. While active travel interventions provide a clear illustration of these dynamics, the methods described in this paper may be similarly useful for other population-level interventions that operate across multiple sectors and disciplines. By foregrounding theory-building, stakeholder perspectives and interactions between interventions and their wider systems, complex systems approaches can help guide data collection and analysis in natural experimental evaluations and support the generation of more context-sensitive and policy-relevant evidence.

### Strengths and limitations

To our knowledge, this is the first paper which applies Alvarado’s method to the early stages of evaluating a population health intervention that was outside of researchers control [6]. We respond to criticism of the existing evidence base by exploring the value of applying this approach to the evaluation of ongoing interventions across multiple sites [5], in addition to engaging stakeholders early in the process to challenge and refine our thinking [45]. This collaborative process expanded our capacity to generate policy-relevant research insights, particularly by considering feedback loops between the drivers of the schemes and their impacts. We hope this will enable us to develop conclusions that resonate with an engaged group of stakeholders, supporting the implementation and sustainability of traffic restriction schemes in the future [6, 11].

It is important to acknowledge that we did not begin with or develop system specific research questions. We applied a complex systems approach to guide our evaluation, rather than conducting a purely systems-focused evaluation [5]. Our initial literature review was relatively focused and specific; in future and with greater resources, we could allocate more time to this step and conduct a formal literature search. We chose a qualitative systems approach, as it aligned best with our research focus. However, we recognise the challenges in interpreting the interaction of multiple feedback loops and the relative strength of relationships within the diagram without using quantitative methods, such as system dynamics modelling.

## Conclusion

We present a case study intended to stimulate discussion, highlight opportunities and challenges and offer insights for future evaluations of population health interventions. We believe this helped us to produce novel research propositions, systems archetypes and a CLD which lay the foundation for an evaluation informed by a complex systems approach. We hope the paper acts as a useful guide for others and our candid report encourages systems novices to embrace systems methods in their own evaluation. We welcome feedback and discussion and hope to leverage this throughout our evaluation process.

## Supplementary Information

Supplementary Material 1.

## Data Availability

All data supporting the findings of this study are available within the paper and its Supplementary Information.
