# Supplementary material for: Applying a complex systems-informed approach to population health interventions: a methodological case-study example of traffic restriction schemes outside schools
Source: BMC Public Health. 2026 May 21;26:2180. doi: 10.1186/s12889-026-27532-9 (PMC13383520; doi:10.1186/s12889-026-27532-9)
Supplement: Supplementary file 1 — Supplementary Material 1. [file 12889_2026_27532_MOESM1_ESM.docx]

Appendices

**Appendix 1:** A map of the underlying system influencing children’s active travel to school focusing on policy, organisational, environmental and individual level influences.


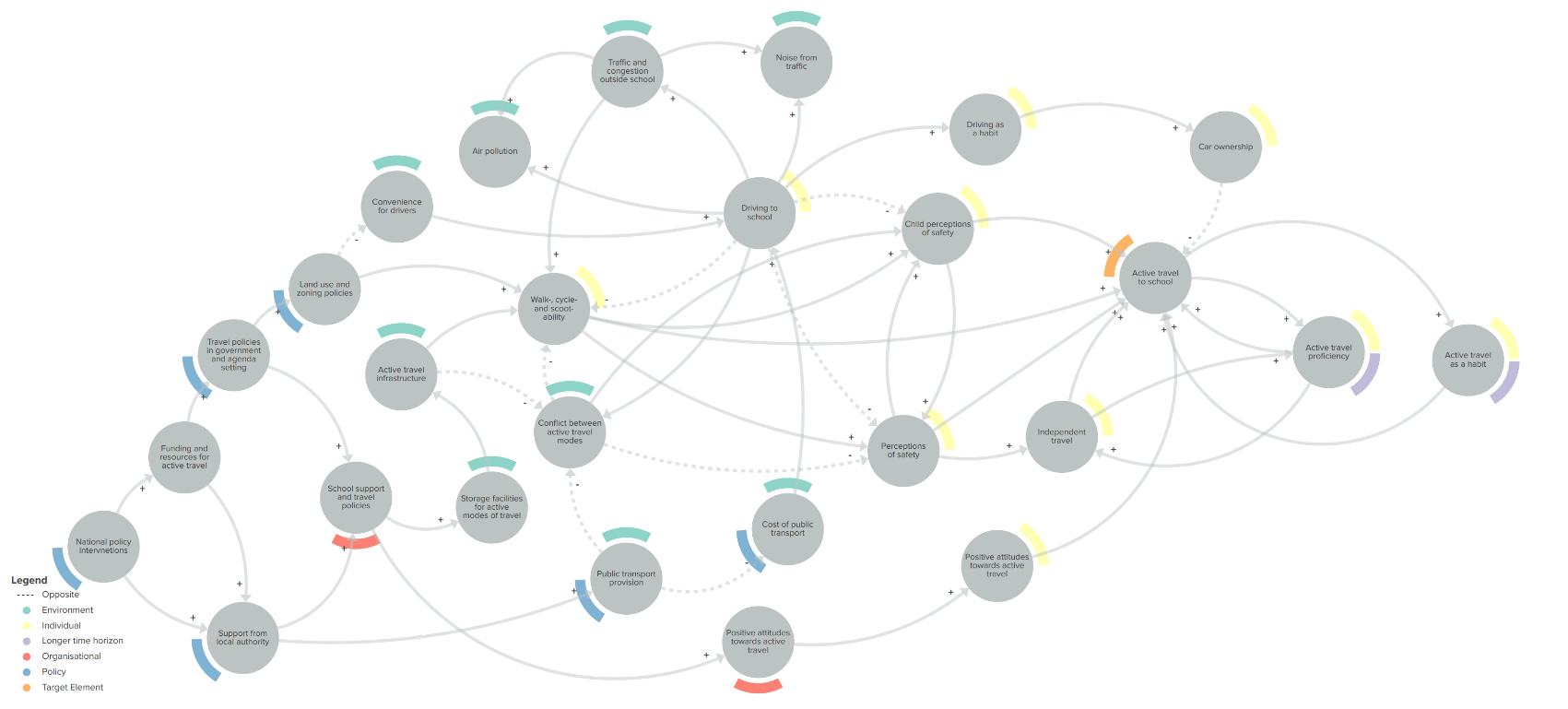


**Appendix 2:** Refined system map with a specific focus on elements which change as a result of traffic restriction schemes


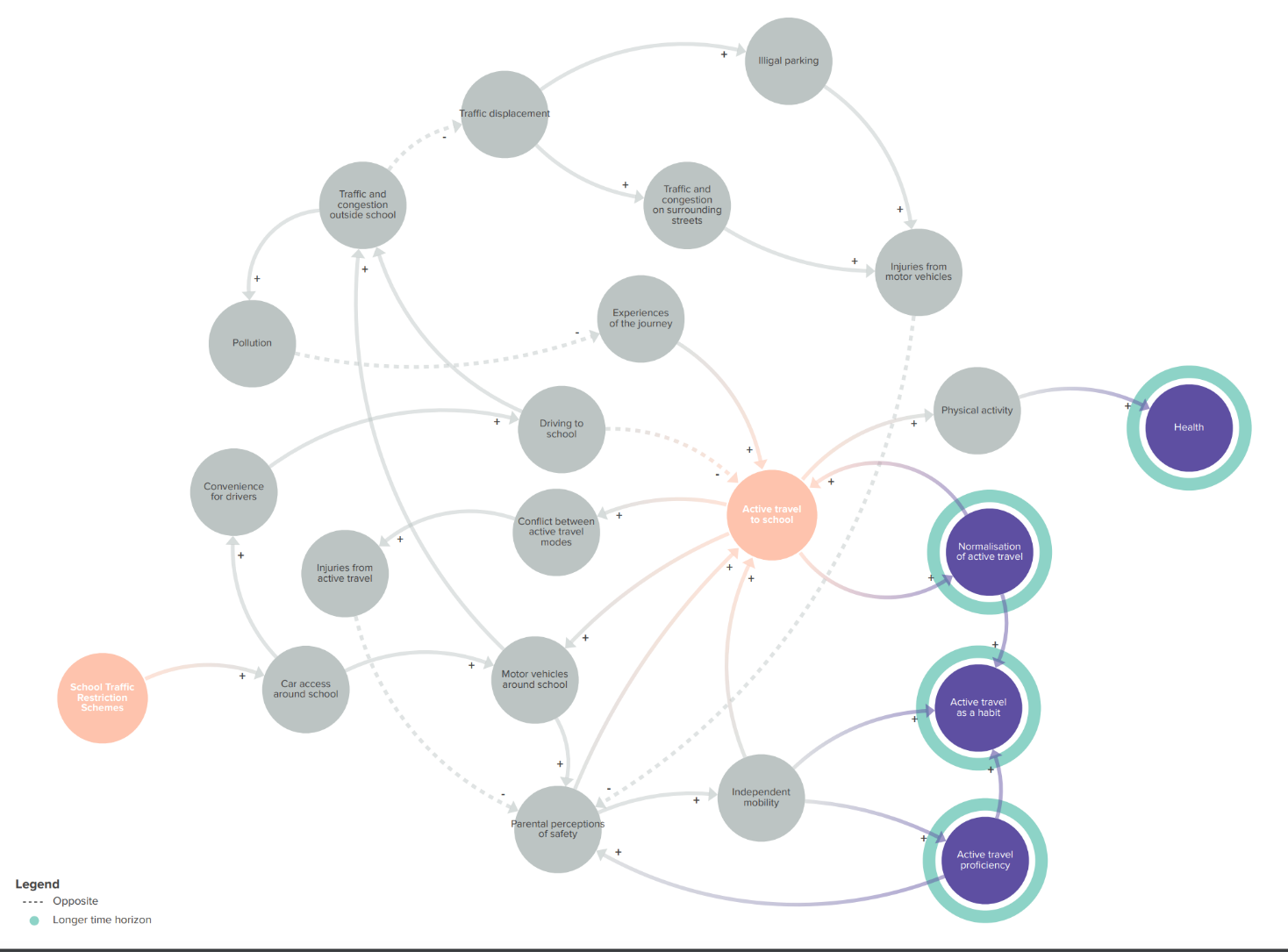


**
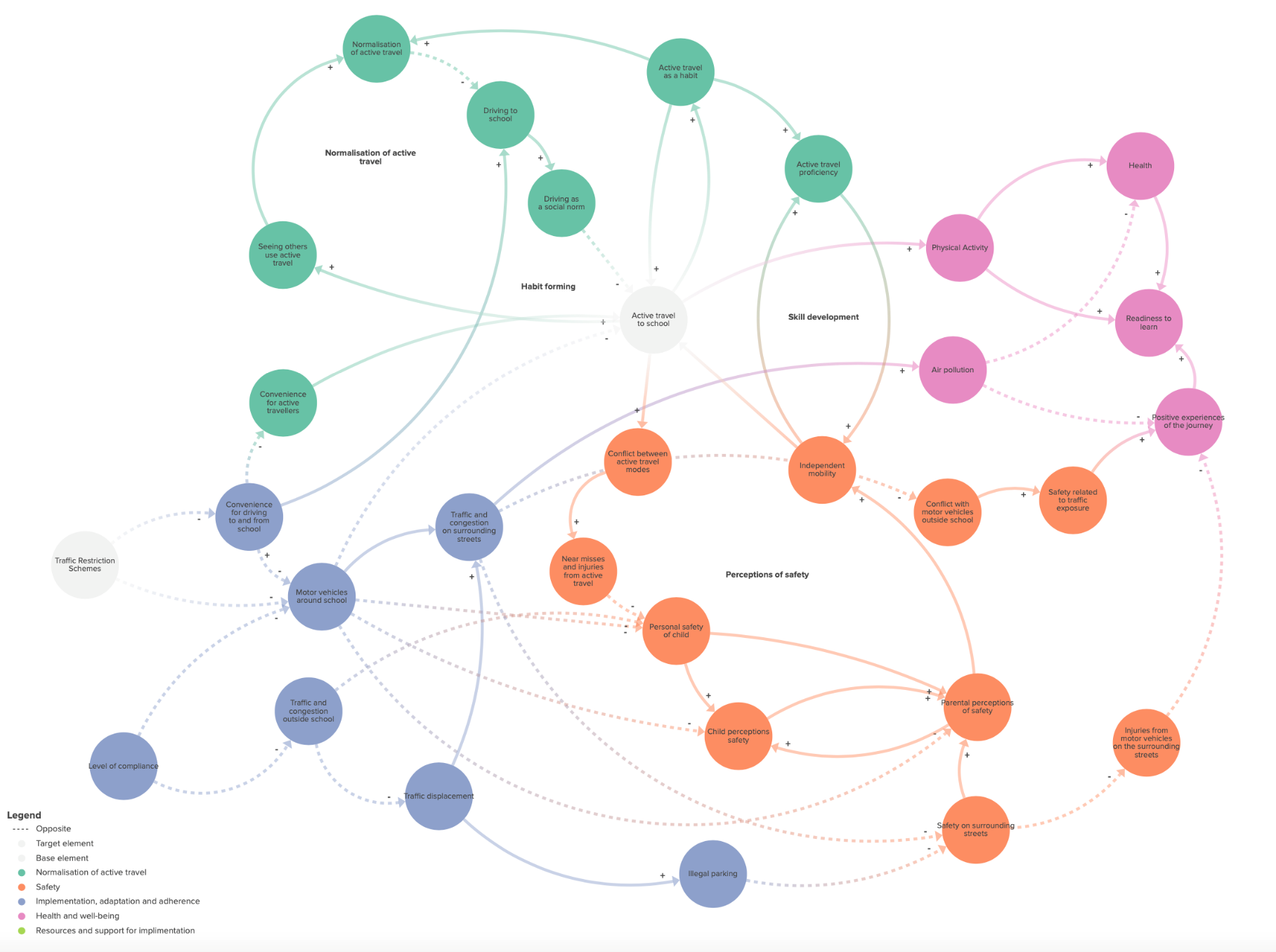
Appendix 3**: Early draft of our combined system maps from steps 1 and 2 into a more detailed causal loop diagram.
